# SDXL model-based optimization for interior design: Data-driven and deep learning methods

**DOI:** 10.1371/journal.pone.0342258

**Published:** 2026-02-04

**Authors:** Xiaofei Zhou, Soohong Kim, Yan Chen

**Affiliations:** 1 School of Art and Design, Dalian Art College, Dalian, Liaoning Province, China; 2 Department of Interior Architecture, Daegu University, Gyeongsan City, North Gyeongsang Province, South Korea; 3 School of Architecture and Fine Art, Dalian University of Technology, Dalian, Liaoning Province, China; Qilu Institue of Technology, CHINA

## Abstract

This study proposes a novel, domain-specific optimization framework for the Stable Diffusion XL (SDXL) model, addressing the critical challenges of structural consistency and aesthetic fidelity in AI-assisted interior design. Unlike generic applications of diffusion models, this research introduces a systematic pipeline integrating automated semantic cleaning with a rigorous hyperparameter optimization strategy. A high-quality, annotated dataset was constructed using a semi-automated YOLO-based filtering process to minimize noise. Furthermore, we established an empirically validated training protocol—combining optimal Dropout rates, L1/L2 regularization, and dynamic learning rates—specifically tuned to preserve the geometric constraints of interior spaces. Experimental results demonstrate that this optimized framework significantly outperforms baseline models, achieving superior Fréchet Inception Distance (FID), Structural Similarity Index (SSIM), and Learned Perceptual Image Patch Similarity (LPIPS) scores, alongside robust CLIP Semantic Alignment. Furthermore, a systematic ablation study confirms that while domain-specific data provides the foundation, our semantic cleaning pipeline and structural regularization are critical for achieving high geometric fidelity, reducing FID by 51.1% compared to the baseline. The study contributes a technically robust methodology for adapting large-scale diffusion models to the specialized requirements of spatial design.

## Introduction

### Research background and motivation

The integration of Artificial Intelligence, particularly through advanced diffusion models, has fundamentally transformed traditional interior design paradigms [1]. However, despite this transformative potential, contemporary AI-assisted systems continue to face significant impediments, particularly regarding the generation of high-fidelity, functionally viable design schematics [2,3]. The emergence of comprehensive interior design datasets, combined with the sophisticated capabilities of the SDXL diffusion model, offers a significant opportunity for innovation in this domain [4]. This study aims to develop an enhanced system that harnesses large-scale interior design data in conjunction with SDXL to produce superior design solutions. Through systematic investigation of data processing methodologies, model optimization techniques, and evaluation frameworks, this research endeavors to advance both the quality and efficacy of AI-assisted interior design while establishing foundational guidelines for AI applications across creative design disciplines.

### Review of related fields

The application of AI in interior design has emerged as a significant research focus, encompassing diverse technological advancements. In image generation, Generative Adversarial Networks (GANs) have been predominant, with Tanasra et al. introducing a conditional GAN-based model for interior design image generation that enables style transfer and layout adjustment [5]. However, GANs encounter challenges in training stability and generation quality. The advent of diffusion models, as demonstrated by Tang et al. [6], has markedly improved the quality and diversity of interior scene synthesis. In data processing, Lee et al. developed an automated annotation system for large-scale interior design datasets [7], while Van et al. investigated various regularization techniques to enhance model generalization. Yousif and Vermisso advanced human-computer collaboration through an interactive AI-assisted interior design system [8].

Although the SDXL model has demonstrated exceptional capabilities in general image generation, its application in specialized domains like interior design remains unexplored. Halle and Hasse emphasized the critical need to incorporate practical functional requirements and construction feasibility in AI-assisted design, aspects often neglected in current research [9]. Despite significant progress, existing literature largely focuses on the direct application of pre-trained models, often overlooking the necessity of domain-specific fine-tuning strategies required for complex spatial tasks.

Crucially, current research lacks a systematic analysis of how hyperparameter configurations influence the structural integrity of generated spaces. Most existing studies apply generic transfer learning without accounting for the non-Euclidean artifacts often introduced by diffusion models in rigid geometric contexts. Addressing this technical gap, our study moves beyond standard model implementation to strictly quantify the impact of regularization and data cleanliness on spatial logic, ensuring the output meets both aesthetic and engineering standards.

### Research objectives and significance

This study aims to optimize model performance and evaluate its practical efficacy through a comprehensive experimental framework. The primary objectives include enhancing model accuracy in design style classification and furniture recognition through the development of high-quality datasets and the implementation of advanced optimization algorithms. The research methodology encompasses a systematic exploration of optimization strategies and hyperparameter configurations to establish optimal training protocols while minimizing computational resources.

This study establishes a semantic-aware optimization framework that integrates an automated data filtration pipeline with a domain-specific fine-tuning protocol. Through the synergistic application of perceptual hashing, object detection, and systematic hyperparameter adjustments, the proposed approach aligns visual fidelity with the structural rigor required for interior architecture. We concurrently introduce a multi-dimensional evaluation standard, augmenting conventional computer-vision metrics (e.g., FID, SSIM, LPIPS) with design-oriented assessments. Ultimately, this study explores potential strategies to reconcile stochastic synthesis with design rigor, hoping to facilitate the broader adoption of generative models in specialized creative domains

## Materials and methods

### Ethical considerations

This study was approved by the authors’ affiliated institution on July 18, 2024. All procedures involving human participants complied with institutional ethical standards and the Declaration of Helsinki and its amendments. Written informed consent was obtained from all participants.

### Data collection

This study utilizes a comprehensive experimental framework to explore optimization methods for interior design by leveraging big data and large models. The data collection process employs web crawling technology to amass diverse image samples from renowned design platforms such as Houzz, Pinterest, and ArchDaily. The dataset then undergoes rigorous quality assessment and annotation, where images are labeled for design attributes using semi-automated tools [10]. Data preprocessing includes normalization and augmentation techniques to enhance model learning efficiency [11]. The study employs SDXL as the foundational model and applies layered fine-tuning strategies tailored to interior design-specific tasks. Various optimization algorithms, including SGD, Adam, and RMSprop, are evaluated. To prevent overfitting, hyperparameter tuning and regularization techniques such as Dropout and L1/L2 regularization are implemented [12,13]. Model performance is assessed through multiple metrics, including accuracy, recall, and F1 score [14]. User satisfaction data are collected through surveys and interviews to assess the practical effectiveness of AI-generated designs. The experimental setup incorporates advanced computational infrastructure to facilitate model training and evaluation. The detailed list of datasets is shown in Table 1.

**Table 1:** Summary of dataset composition.

Category Type	Specific Category	Primary Source(s)	Filtered Pool	Core Images	Training Images
Design Style Focus	Modern Style	Houzz, ArchDaily, Pinterest	525	150	480
	Minimalist Style	ArchDaily, Pinterest	385	110	352
	Scandinavian Style	Pinterest, Houzz	350	100	320
	Industrial Style	Pinterest, ArchDaily	280	80	256
	Rustic Style	Houzz, Pinterest	210	60	192
	Vintage Style	Houzz, Pinterest	175	50	160
	European Style	Houzz, Pinterest	210	60	192
	Chinese Style	Houzz, Pinterest	245	70	224
Subtotal	N/A	N/A	2380	680	2176
Furniture Type Focus	Sofa	All Platforms	175	50	160
	Table/ Desk	All Platforms	175	50	160
	Chair	All Platforms	140	40	128
	Lighting	All Platforms	75	50	160
	Bed	All Platforms	105	30	96
	Storage/ Cabinet	All Platforms	210	60	192
	Other/ Decor	All Platforms	210	40	128
Subtotal	N/A	N/A	1190	320	1024
TOTAL	N/A	N/A	3570	1000	3200

*Note: Training images are calculated based on an 80% training split followed by a 4x offline* augmentation.

### Experimental framework and environment

Hardware environment: Intel Core i7-13900KF CPU, NVIDIA GeForce RTX 4090 GPU, 64 GB DDR5 RAM, and a 2 TB NVMe SSD.

Software environment: Windows 11 Professional; Anaconda (Python 3.11.11); CUDA Toolkit 12.6; PyTorch 2.2.0 with xFormers for memory‑efficient attention; TensorFlow 2.11; diffusers 0.24.0; DreamBooth-for-Diffusion for large-model training; Scrapy 2.8 for data acquisition; Stable Diffusion WebUI and ComfyUI for model testing; SPSS 27.0 for statistical analysis; and custom Python scripts using OpenCV and Pillow for image-generation model validation.

### Data processing pipeline

To establish a robust foundation for model training, we implemented a systematic data processing pipeline comprising three phases: acquisition, refinement, and preparation. The comprehensive workflow is illustrated in S1 Fig.

### Distributed acquisition and ethical compliance

A distributed web crawling system was developed based on the Scrapy framework to aggregate large-scale interior design imagery from Houzz, Pinterest, and ArchDaily. To maintain ethical compliance and system stability, the architecture utilized dynamic proxy pools and strictly adhered to “polite crawling” protocols (Core code in **Appendix A in** S1 File).

Following acquisition, a multi-stage cleaning protocol was implemented to filter noise. First, Perceptual Hashing (pHash) was applied to eliminate duplicate images. Second, an automated quality assessment filter removed images with low variance (blur) or poor exposure using histogram equalization. Finally, a pre-trained YOLO model [15] was employed to filter out irrelevant content (e.g., floor plans, exterior shots) that did not contain interior spatial elements. The efficiency of this filtration process is summarized in Table 2 (Core code in **Appendix B in** S1 File).

**Table 2:** Data cleaning and filtration statistics.

Processing Stage	Method	Input Count	Output Count	Retention Rate
Stage 1: Raw Acquisition	Distributed Scrapy Crawler	5000	5000	100%
Stage 2: Deduplication	Perceptual Hashing (pHash)	5000	4215	84.3%
Stage 3: Quality Control	Laplacian Variance/ Contrast	4215	3850	91.3%
Stage 4: Semantic Filtering	YOLO Object Detection	3850	3570	92.7%
Total Efficiency	End-to-End Pipeline	5000	3570	71.4%

The efficiency of this filtration process is summarized in Table 2. From the filtered pool of 3570 images, a balanced subset of 1,000 core images was manually currated to ensure even distribution across design styles and furniture categories.

### Hierarchical annotation and preprocessing

Accurate semantic labeling is critical for conditional image generation. We adopted a human-in-the-loop approach where a fine-tuned YOLO model provided preliminary labels, which were subsequently verified and corrected via a collaborative interface. A hierarchical multi-label taxonomy was defined to capture Design Style, Furniture Layout, and Spatial Function (see Table 3; Core code in **Appendix C in** S1 File).

**Table 3:** Hierarchical annotation taxonomy.

Level 1: Spatial Function	Level 2: Design Style	Level 3: Key Furniture Elements (Multi-label)
Living Room	Modern, Minimalist	Sofa, Coffee Table, TV Unit, Floor Lamp
Kitchen/ Dining	Industrial, Scandinavian	Dining Table, Pendant Light, Cabinet, Bar Stool
Bedroom	European, Rustic	Bed, Wardrobe, Nightstand, Rug
Bathroom	Vintage, Chinese	Vanity, Mirror, Bathtub, Shower Enclosure
Study/ Office	Modern, Industrial	Desk, Ergonomic Chair, Bookshelf, Task Lighting

### Data normalization and augmentation

Prior to model ingestion, images underwent rigorous normalization and augmentation [16]. All images were resized to 512×512 with zero-padding to preserve aspect ratio. Pixel values were normalized to the range [0,1] to stabilize gradient descent. To enhance model robustness and prevent overfitting on the limited dataset, a stochastic data augmentation strategy was applied during training, utilizing geometric and photometric transformations detailed in Table 4 (Core code in **Appendix D in** S1 File).

**Table 4:** Data augmentation hyperparameters.

Augmentation Technique	Probability (*p*)	Parameter Range/ Configuration	Purpose
Random Horizontal Flip	0.5	–	Invariance to lateral orientation
Random Rotation	0.3	Degrees ∈[−10,10]	Robustness to camera angle variations
Color Jitter	0.4	Brightness ±0.2, Contrast ±0.2	Adaptability to lighting conditions
Random Crop & Resize	0.5	Scale ∈[0.8,1.0]	Focus on local furniture details
Gaussian Noise	0.2	σ∈[0.01,0.05]	Simulation of sensor noise/ texture

### Model selection and fine-tuning

The experimental design treated model selection as a key determinant of accuracy and robustness. We chose the SDXL model for its broad adoption in image generation, strong community support, and compatibility with available laboratory hardware, enabling efficient computation during both training and inference despite its substantial parameter count.

To achieve efficient parameter updates on the SDXL architecture while adhering to hardware constraints, we implemented the Low-Rank Adaptation (LoRA) technique within the DreamBooth framework. Unlike full parameter fine-tuning, LoRA freezes the pre-trained model weights and injects trainable rank decomposition matrices into the distinct layers of the strictly attention mechanism. This allows for the “layered fine-tuning” of style-specific features without catastrophic forgetting. Training was conducted using Mixed Precision (fp16) to optimize memory usage and throughput.

To ensure reproducibility, the precise training configuration derived from our optimization experiments is consolidated in Table 5.

**Table 5:** Optimal training configuration for reproducibility.

Parameter Category	Specific Setting	Value/ Type
Base Model	Version	SDXL 1.0 Base
	VAE Precision	fp16 (Fixed)
Fine-Tuning Method	Technique	LoRA (DreamBooth)
	LoRA Rank (Dimension)	128
	LoRA Alpha	128
	Target Modules	UNet Attention Layers (to_k, to_q, to_v, to_out)
Optimization	Optimizer	AdamW
	Optimizer Parameters	$\beta_{\text{1}}=\text{0}.\text{9},\beta_{\text{2}}=\text{0}.\text{999}$, Weight Decay=1e−2
	Precision	Mixed Precision (fp16)
	Gradient Accumulation	1 step
Hyperparameters	Global Batch Size	16
	Learning Rate	1e−4 (Peak) with Cosine Annealing
	Epochs	140 (Early Stopping triggered)
	Regularization	Dropout (p=0.2), L1 Norm (λ=0.01)
Data Handling	Resolution	512×512 (Center Crop)
	Noise Offset	0.1 (To improve contrast)

### Hyperparameter optimization

#### Learning rate adjustment.

A dynamic method for adjusting the learning rate by gradually reducing it during training, enhancing model convergence and stability. The experiment used the cosine annealing strategy, described by the following formula (1) [17].


$$\begin{array}{c} {}*20c\eta_{t}=\eta_{min}+\frac{\text{1}}{\text{2}}\left(\eta_{max}-\eta_{min}\right)\left(1+cos\left(\left(\frac{t}{T}\right)\pi \right)\right) \end{array}$$ (1)

In this context, $\eta_{\text{m}}$ and $\eta_{max}$ present the minimum and maximum learning rates. The variable. t refers to the current iteration number, and T is the total number of iterations. Initially setting a larger learning rate helps in rapidly searching for the global optimum, while gradually reducing it in later training stages facilitates fine-tuning of model parameters.

Warm Restarts is a training strategy involving the periodic resetting of the learning rate to a relatively high initial value, followed by its gradual reduction. This approach can assist the model in escaping local optima and enhance its global search capability. The specific learning rate schedule employed in this strategy is described by Formula (2) [18].


$$\begin{array}{c} {}*20c\eta_{t}=\eta_{min}+\frac{\text{1}}{\text{2}}\left(\eta_{max}-\eta_{min}\right)\left(1+cos\left(\left(\frac{t_{mod}}{T_{cur}}\right)\pi \right)\right) \end{array}$$ (2)

In this context, $t_{mod}$ is the remainder of the current iteration number divided by the current cycle length, and $T_{cur}\;$is the current cycle length. Each time a cycle ends, the cycle length increases, allowing for more fine-grained adjustment of model parameters in subsequent training.

Cyclical Learning Rates involves cyclically varying the learning rate between two boundaries and continuously adjusting it during training to enhance model robustness and generalization ability, described by the following formula (3) [19].


$$\begin{array}{c} {}*20c\eta_{t}=\eta_{min}+\left(\eta_{max}-\eta_{min}\right)\cdot \;triangular\left(t\right) \end{array}$$ (3)

In this context, the triangular(t) function is a triangular function used to cyclically vary the learning rate between $\eta_{min}$ and $\eta_{max}$. This approach allows for continuous exploration of new parameter spaces during training, thereby improving model performance.

The impact of different learning rate schedules on model training is illustrated in S2 Fig.

We configured and tuned the learning rate to promote rapid, stable convergence: Initial learning rate $\eta_{max}=\text{0}.\text{001}$, $\eta_{min}=\text{0}.\text{00001}$. a schedule period T of 10 epochs with an initial warm-restart cycle of 10 epochs, and cyclical boundaries spanning [0.00001, 0.001]. These settings yielded fast, stable training convergence; detailed parameters are provided in Table 6.

**Table 6:** Learning rate settings.

Strategy name	Initial learning rate	Minimum learning rate	Schedule period/steps	Description
Cosine annealing	0.001	0.00001	10 epochs	The learning rate gradually decreases and stabilizes within each cycle.
Warm restarts	0.001	0.00001	10 epochs	Resets the learning rate at the end of each cycle, with increasing cycle length.
Cyclical learning rates	0.001	0.00001	5 epochs	Learning rate cycles between two bounds to improve generalization capability.

### Batch size selection

Batch size, a key deep-learning hyperparameter, specifies how many samples are used per iteration to compute gradients and update parameters [20]. Larger batches improve parallel efficiency and speed convergence but demand more memory and can drive convergence toward sharper minima with weaker generalization. Smaller batches add gradient stochasticity that can aid generalization by favoring flatter minima and escaping poor optima, yet they slow training and may become unstable if too small. Choosing batch size thus balances computational efficiency, memory limits, and generalization.

To investigate the impact of batch size on SDXL model training, experiments were conducted using batch sizes of 4, 8, 16, and 32. Given the dataset’s limited size (3200 images), larger batch sizes were expected to increase the risk of rapid overfitting. The dataset was partitioned into training, validation, and test sets using an 8:1:1 ratio. Model performance was evaluated using the FID and CLIP Score. Additionally, the training time for each batch size was recorded. S3 Fig illustrates the training loss curves for each batch size configuration.

From the experimental results, it is evident that for a small dataset of 3200 images, a batch size of 16 yielded the best performance in terms of FID and CLIP Score. Although a batch size of 32 resulted in the fastest training speed, the FID value slightly increased, indicating a potential decrease in generalization ability. Considering model performance, training time, and memory consumption, a batch size of 16 was ultimately chosen for training the SDXL model.

### Determining epochs

An epoch represents one complete pass through the entire training dataset by the model. The number of epochs, a key hyperparameter, dictates the total duration of the training process. Selecting an appropriate number of epochs is crucial for balancing effective model fitting with generalization ability. Insufficient epochs may lead to underfitting, where the model fails to adequately capture underlying data patterns. Conversely, excessive epochs can cause overfitting, resulting in the model memorizing the training data, including noise, which degrades performance on unseen test data.

To determine the optimal number of epochs for training the SDXL model, a series of experiments were conducted using different epoch counts (50, 100, 150, 200). The dataset was the same as the batch size experiment, split into training, validation, and test sets at an 8:1:1 ratio, with a fixed batch size of 16. The evaluation metrics remained FID and CLIP Score, with training time recorded for each epoch count, as shown in S4 Fig.

### Regularization techniques

Dropout is a widely used regularization technique that randomly deactivates neurons and their connections during training. In each iteration, neurons are retained with probability p or dropped with probability 1-p. This prevents neuron co-adaptation and promotes robust, generalizable features by forcing the model to learn with varied network architectures. Effectively, Dropout trains an ensemble of shared-weight ‘thinned’ networks, enhancing model generalization [21].

To systematically study the effect of Dropout on SDXL model training, the experiment-controlled variables. Different dropout rates (p = 0.1, 0.2, 0.3, 0.4, 0.5) were tested while keeping other hyperparameters (such as batch size, learning rate, and epochs) constant. The dataset maintained an 8:1:1 split ratio for training, validation, and test sets, using FID and CLIP Score as the main performance evaluation metrics. By comparing model performances at different Dropout rates, the experiment aimed to determine the optimal Dropout rate to balance model complexity and generalization ability. The experimental results are shown in S5 Fig.

Experimental results demonstrate that incorporating Dropout effectively mitigates overfitting and enhances model generalization. As shown in S5 Fig, the model attained optimal FID and CLIP Scores on the validation set with a Dropout rate of 0.2. This suggests that moderate Dropout provides effective regularization. Conversely, both excessively high and low Dropout rates appear detrimental to performance. Specifically, an insufficient Dropout rate offers limited regularization, whereas an excessive rate risks hindering the model’s learning capacity, potentially leading to underfitting. Consequently, a Dropout rate of 0.2 was selected for training the SDXL model.

L1 and L2 regularization curb overfitting by adding weight penalties to the loss, constraining model complexity [22]. L1 (Lasso) uses the L1 norm to promote sparsity, driving unimportant weights to zero and enabling implicit feature selection. L2 (Ridge) uses the squared L2 norm to shrink weights uniformly without zeroing them, reducing reliance on any single feature and improving robustness. Both yield smaller weights, smoother mappings, and better generalization.

To explore the impact of L1 and L2 regularization on the SDXL model’s training, a series of comparative experiments were conducted. The experiments used different regularization strengths (λ = 0.001, 0.01, 0.1) and compared model performance with L1, L2, and without regularization. The dataset maintained an 8:1:1 split for training, validation, and test sets. Batch size and epochs were fixed at 16 and 140, respectively. The performance evaluation metrics were FID and CLIP Score, as shown in S6 Fig.

Early stopping regularizes training by monitoring performance on a validation set and halting when improvement stalls or reverses [23]. Training loss may keep decreasing, but validation performance typically peaks before degrading, signaling overfitting. Stopping at this point prevents further overfitting and preserves the parameters associated with the best validation performance.

To verify the effectiveness of Early Stopping, the same training data and hyperparameter settings (including batch size, number of epochs, optimization algorithm, and L1 regularization) were used to train the SDXL model. The FID and CLIP scores were recorded on the training and validation sets at the end of each epoch. The patience was set to 10, meaning training would stop if the FID value on the validation set did not decrease for 10 consecutive epochs. By observing trends in the loss curves and performance metrics, the experiment can determine whether Early Stopping effectively prevents overfitting and helps select the optimal training stop point. The results are shown in S7 Fig.

The FID score on the training set decreased throughout training, whereas the validation set FID reached its minimum value around epoch 120 and subsequently increased. This pattern indicates the onset of overfitting after epoch 120. Accordingly, Early Stopping terminated training at epoch 140, thereby preventing substantial further overfitting.

## Results

### Performance evaluation

#### Accuracy.

To comprehensively evaluate the performance of the SDXL model following data augmentation and hyperparameter optimization, its classification capabilities were assessed on a reserved test set using three standard metrics: Accuracy, Recall, and F1-score [24]. These metrics collectively provide a robust measure of performance, particularly for the target tasks of design style classification and furniture recognition. Accuracy reflects the overall proportion of correct classifications, Recall quantifies the model’s ability to identify all relevant instances within each class, and the F1-score offers a balanced assessment by calculating the harmonic mean of Precision and Recall, making it especially informative in cases of potential class imbalance.

The trained SDXL model was evaluated using the test set, and the accuracy, recall, and F1 scores were calculated for both design style classification and furniture recognition tasks. The results are shown in Table 7.

**Table 7:** Performance of the SDXL model in design tasks.

Task	Accuracy	Recall	F1 score
Design style classification	0.85	0.82	0.83
Furniture recognition	0.92	0.88	0.90

Confusion matrices were generated to provide a clear visual representation of classification performance. S8 Fig-S9 Fig depict these matrices for the design style classification and furniture recognition tasks, respectively, illustrating the model’s prediction accuracy across the different categories.

Analysis of Table 7 and the confusion matrices reveals that the SDXL model achieved high Accuracy, Recall, and F1-scores across both design style classification and furniture recognition tasks. These results indicate the model’s capacity to effectively learn distinguishing features within interior design images, enabling accurate classification of various design styles and furniture types. Notably, performance was slightly superior for the furniture recognition task compared to design style classification. This difference may stem from the typically more distinct visual characteristics of furniture items, potentially facilitating recognition by the model.

### Image quality assessment

Beyond classification accuracy, the quality of generated images was evaluated, a critical consideration for interior design applications requiring visual fidelity and aesthetic appeal that accurately reflect specific styles and furniture. We assessed model performance with a multidimensional suite tailored to interior design—FID, SSIM, LPIPS [25], and CLIP Score. FID quantifies the distance between feature distributions of real and generated images, while SSIM captures low-level structural similarity in luminance and contrast. LPIPS measures perceptual similarity in the deep feature space of a pre-trained VGG network [26], where lower values indicate outputs that align more closely with human visual judgment. CLIP Score evaluates semantic consistency by computing the cosine similarity between image and text embeddings, ensuring that user-specified styles (e.g., “Industrial”) are faithfully reflected in the generated designs. [27].

The experiment used a pre-trained Inception V3 network to extract image features and calculate FID values between the generated and real images [28]. To evaluate style transfer effectiveness, SSIM values were also calculated between generated images and their corresponding target style images. The results are shown in Table 8.

**Table 8:** Comprehensive image quality assessment.

Style	FID ↓	SSIM ↑	LPIPS ↓ (Perceptual)	CLIP Score ↑ (Semantic)
Modern	18.5	0.88	0.142	0.312
European	22.3	0.85	0.165	0.295
Chinese	19.7	0.87	0.151	0.301
Minimalist	17.2	0.89	0.128	0.325

Experimental results demonstrate robust performance across all four metrics. The low LPIPS scores (<0.17 across all styles) confirm that the generated images possess high perceptual realism, avoiding the “waxy” or artifact-heavy textures common in unoptimized models. Furthermore, the consistently high CLIP Scores (>0.29) validate the model’s semantic accuracy, ensuring that distinct stylistic elements—such as the ornate details of European design or the clean lines of Minimalism—are correctly rendered according to the text prompts. The Minimalist style achieved the best overall performance (LPIPS 0.128, CLIP 0.325), likely due to its reduced visual complexity which aligns well with the model’s latent biases.

### Inference speed analysis

Model inference speed is a critical performance factor in practical applications demanding real-time user interaction, such as interactive design tools and virtual reality environments [29]. To quantify the efficiency gains resulting from the applied optimization strategies, the inference speed of the optimized SDXL model was evaluated and compared against that of the baseline model.

Inference speed tests were performed on both models using identical hardware and a test set comprising 100 diverse interior design images. Performance was measured by the average processing time per image and throughput, quantified as Images Per Second (IPS). The optimized model employed the previously determined optimal hyperparameters (batch size: 16, epochs: 140, optimizer: Adam, regularization: L1 with strength 0.01), while the baseline model utilized default settings. Results are presented in Table 9.

**Table 9:** Inference speed comparison of the SDXL model.

Model	Average Inference Time (ms) ↓	Images Per Second (IPS) ↑
Base Model	85	11.8
Optimized Model	42	23.8

The data presented in Table 9 demonstrate a significant improvement in the inference speed of the optimized SDXL model. Relative to the baseline model, the optimized model achieved an approximate 50% reduction in average inference time and a corresponding ~100% increase in IPS. These findings confirm that the applied optimization strategies yielded substantial improvements not only in model performance metrics but also in inference efficiency. Consequently, the increased processing speed of the optimized model enhances its suitability for real-time applications. This enhanced efficiency is primarily attributed to the optimized hyperparameters, particularly the selected batch size, which leverages GPU parallel processing capabilities more effectively.

### User study protocol and statistical methodology

To rigorously assess the semantic and functional quality of the optimized SDXL model, we implemented a double-blind A/B test. One hundred participants were recruited and stratified into General Users (N = 50) and Professional Interior Designers (N = 50). The sample size was informed by a G*Power analysis, which indicated that at least 42 participants per group were required to detect a medium effect (Cohen’s d = 0.5) with 80% power at α = 0.05.

Participants viewed paired images addressing the same design brief—one produced by our optimized model and the other by a human designer using standard CAD tools—without disclosure of source to either participants or administrators. Each design was rated on 5-point Likert scales across four dimensions: Aesthetics (visual appeal and stylistic consistency), Functionality (layout rationality and ergonomics), Innovation (creativity and uniqueness), and Overall Satisfaction.

Group means for AI- versus human-generated designs were compared using independent-samples t-tests, with statistical significance set at p < 0.05. Results are summarized in S10 Fig

### Ablation study

We conducted a systematic ablation to disentangle the contributions of our two modules: a semantic-aware data pipeline and domain-specific structural regularization. Four configurations were compared: M1, the SDXL 1.0 baseline without domain adaptation; M2, SDXL fine-tuned on the raw scraped dataset (~5,000 images); M3, SDXL fine-tuned on 3200 images curated by our semantic-aware cleaning pipeline without structural regularization; and M4, our full framework combining semantic cleaning, LoRA fine-tuning, and joint L1/L2-Dropout regularization.

From M1 to M2, the CLIP score improves from 0.245 to 0.298, underscoring the importance of domain-specific data. Yet M2 yields the highest FID (28.4), likely due to semantic noise in the unfiltered corpus (e.g., floor plans and low-quality shots). Introducing the cleaning pipeline (M3) lowers FID to 21.5, indicating that data quality outweighs quantity. Adding structural regularization (M4) further delivers the best SSIM (0.89) and the lowest FID (17.2), showing that while cleaning enforces stylistic fidelity, our regularization is pivotal for preserving geometric logic and suppressing the “waxy,” non-Euclidean artifacts typical of generative spatial design (S11 Fig).

## Discussion

This study aimed to bridge the gap between general-purpose diffusion models and the specialized requirements of interior design. By proposing a domain-specific optimization framework, we addressed critical challenges in data quality, structural consistency, and evaluation validity. The following analysis interprets our experimental findings in the context of the primary research contributions.

### Validation of the semantic-aware data pipeline

The first contribution of this study was the construction of a semantic-aware dataset pipeline to mitigate the “noisy label” issue prevalent in scraped data. Our experimental results on classification tasks (Table 7) strongly corroborate the efficacy of this approach. The model achieved high F1-scores for both design style (0.83) and furniture recognition (0.90). This superior performance indicates that the automated filtering utilizing fine-tuned YOLO models [15] successfully removed ambiguous or irrelevant imagery. Unlike standard datasets where noise leads to feature degradation, our rigorous cleaning protocol ensured that the SDXL model learned distinct, high-fidelity stylistic representations, validating the importance of data normalization strategies emphasized in data-driven design [11].

### Efficacy of domain-specific optimization strategies

The second contribution focused on identifying a hyperparameter configuration specifically tuned for the geometric constraints of interior space. The quantitative results (Table 8) demonstrate that our optimized framework significantly outperforms the baseline. The achievement of low FID (17.2) and high SSIM (0.89) scores confirms that the fine-tuned model generates images with high perceptual realism and structural fidelity [25].

These results directly validate our hypothesis regarding the combinatorial effect of optimization techniques. Specifically, the use of Cosine Annealing and Warm Restarts [17,18] facilitated rapid convergence, while Cyclical Learning Rates [19] helped the model escape local optima. Furthermore, the selection of an optimal batch size (16) adhered to the generalization principles outlined by He et al. [20]. Critically, the implementation of a moderate Dropout rate (p=0.2) [21] combined with L1/L2 regularization [22] and Early Stopping [23] proved essential in preventing the model from overfitting to high-frequency noise, ensuring that generated layouts maintained logical spatial structures rather than just memorizing training textures.

### The Necessity of an integrated evaluation framework

Our third contribution was the establishment of a holistic evaluation framework. The A/B testing results (S10 Fig) revealed a significant dichotomy: while general users rated AI designs comparably to human designs (p>0.05), professional designers identified a statistically significant deficit in Functionality (p<0.001).

This finding supports the concerns raised by Halle and Hasse [9] regarding the neglect of functional feasibility in AI design. It demonstrates that computational metrics like FID are insufficient for assessing “usability” in professional domains. By exposing this “practicality gap,” our study reinforces the argument by Yousif and Vermisso [8] for human-in-the-loop systems, proving that AI models currently excel at aesthetic suggestion but require professional oversight for functional implementation.

### Mechanisms of modular improvement

The ablation results reveal a critical synergy between data purity and structural constraints. The transition from M2 to M3 (resulting in a 24.3% FID reduction) demonstrates that semantic noise—such as floor plans and non-interior shots—directly degrades the model’s ability to learn stylistic features. However, even with clean data (M3), the model remains prone to “hallucinating” non-Euclidean geometries. The final integration of structural regularization in M4 solves this by enforcing weight sparsity and preventing overfitting to high-frequency textures, thereby shifting the model’s focus toward the underlying geometric logic. This confirms that for interior design, visual quality (FID) is driven by data cleaning, while functional viability (SSIM) is sustained by structural regularization.

### Limitations and future work

This study, while promising, has several limitations. The curated dataset remains modest in scale—approximately 3200 images—and lacks coverage of highly specialized architectural idioms such as parametricism or deconstructivism, which constrains generalization to styles underrepresented in training. The generative backbone operates purely in 2D pixel space [30]; images can appear visually coherent yet harbor subtle perspective inconsistencies or physically untenable geometries that impede direct translation to 3D BIM [31]. Professional assessment also indicates difficulty with functional and ergonomic logic, including kitchen work triangles and ADA-compliant layouts.

Future work will incorporate ControlNet to impose explicit structural constraints and investigate text-to-3D pipelines to close the gap between 2D concept synthesis and construction-ready documentation. These directions aim to enhance geometric fidelity, functional plausibility, and stylistic breadth without sacrificing the visual quality achieved in the present framework.

## Conclusions

This study presents a domain-specific optimization framework for the SDXL model, successfully reconciling the tension between generative creativity and the rigorous constraints of interior design. By constructing a high-fidelity, semantic-aware dataset and implementing a fine-tuned training protocol (incorporating LoRA, optimal Dropout, and regularization), we achieved significant improvements in both visual fidelity (FID 17.2) and semantic alignment (CLIP Score 0.325). Rigorous ablation testing validates that the success of this framework is not merely additive but synergistic: the semantic-aware data pipeline ensures stylistic accuracy, while the domain-specific regularization protocol provides the structural rigidity necessary for interior architecture. This modular approach provides a scalable template for adapting general-purpose generative AI to other high-precision design disciplines.

The practical significance of this work extends beyond algorithmic metrics to the professional design workflow. The optimized model’s 100% increase in inference speed renders it a viable tool for real-time human-AI collaboration. Designers can utilize this system for rapid iterative prototyping in the early conceptual phases, instantly visualizing complex stylistic requirements (e.g., “Minimalist layout with Industrial lighting”) during client consultations. This capability significantly reduces the time-intensive overhead of traditional manual rendering, allowing designers to focus on high-level spatial problem-solving rather than technical visualization.

Despite strong performance, the gap in functional practicality underscores the need for further development. We will pursue two directions: first, integrating structural control through ControlNet with edge-detection guidance (e.g., Canny or depth maps) to suppress “hallucinated” impossible geometries and enforce strict adherence to architectural blueprints during diffusion; second, advancing 2D-to-3D interoperability by generating 3D-consistent assets—using NeRF or Gaussian Splatting—that can be imported directly into BIM environments, thereby linking conceptual image generation to constructible engineering documentation.

## Supporting information

S1 Fig Comprehensive workflow. (TIF)

S2 Fig Learning rate adjustment strategy curves. (TIF)

S3 Fig Training loss curves for different batch sizes. (TIF)

S4 Fig Loss curves on training and validation sets for different epoch counts. (TIF)

S5 Fig Loss curves on training and validation sets for different dropout rates. (TIF)

S6 Fig Training and vaildation loss with different regularization methods. (TIF)

S7 Fig FID value change curves for training and validation sets. (TIF)

S8 Fig Confusion matrix for design style classification. (TIF)

S9 Fig Confusion matrix for furniture recognition task. (TIF)

S10 Fig Professional Designers Evaluation: AI vs Human. (TIF)

S11 Fig Ablation Study: Contribution of Proposed Modules. (TIF)

S1 File Appendix A-D. (DOCX)
