# Supplementary figures and images for: SDXL model-based optimization for interior design: Data-driven and deep learning methods
Source: PLoS One. 2026 Feb 4;21(2):e0342258. doi: 10.1371/journal.pone.0342258 (PMC12871978; doi:10.1371/journal.pone.0342258)

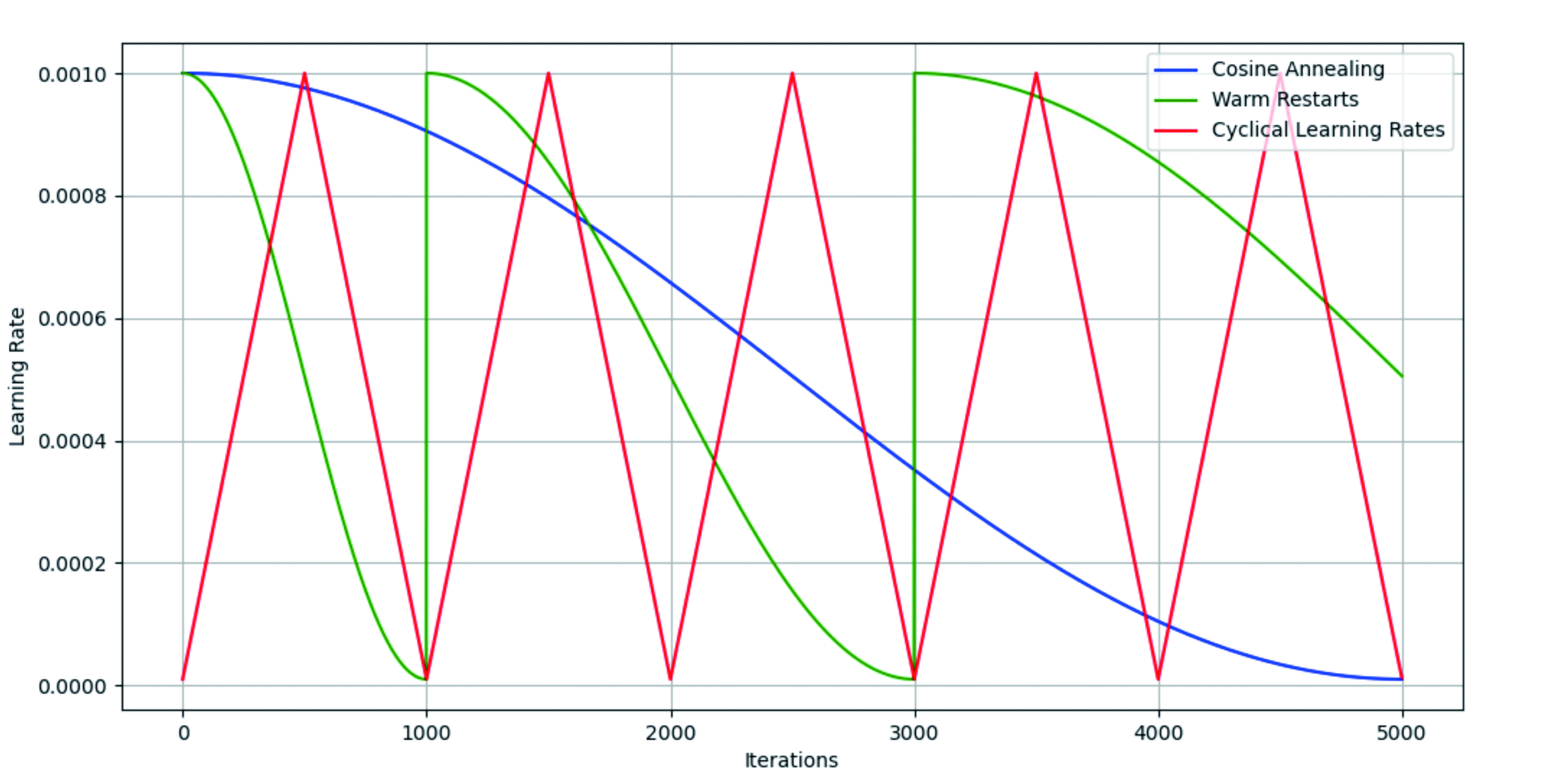

Supplement: S2 Fig — (TIF) [file pone.0342258.s002.tif]

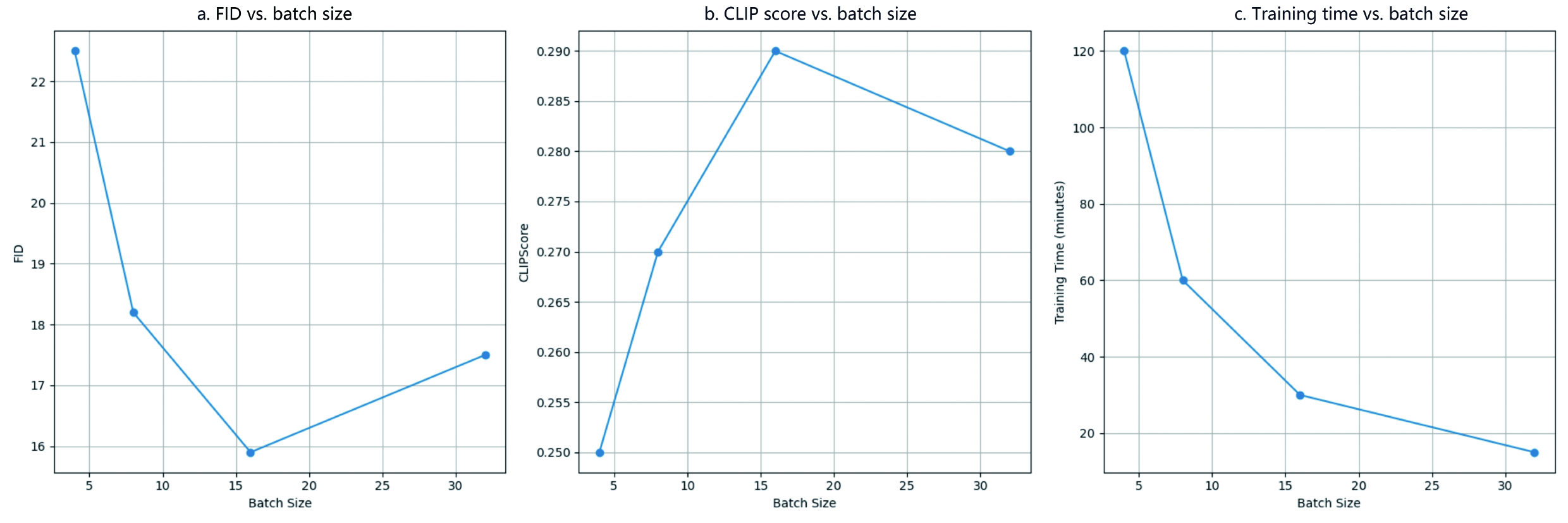

Supplement: S3 Fig — (TIF) [file pone.0342258.s003.tif]

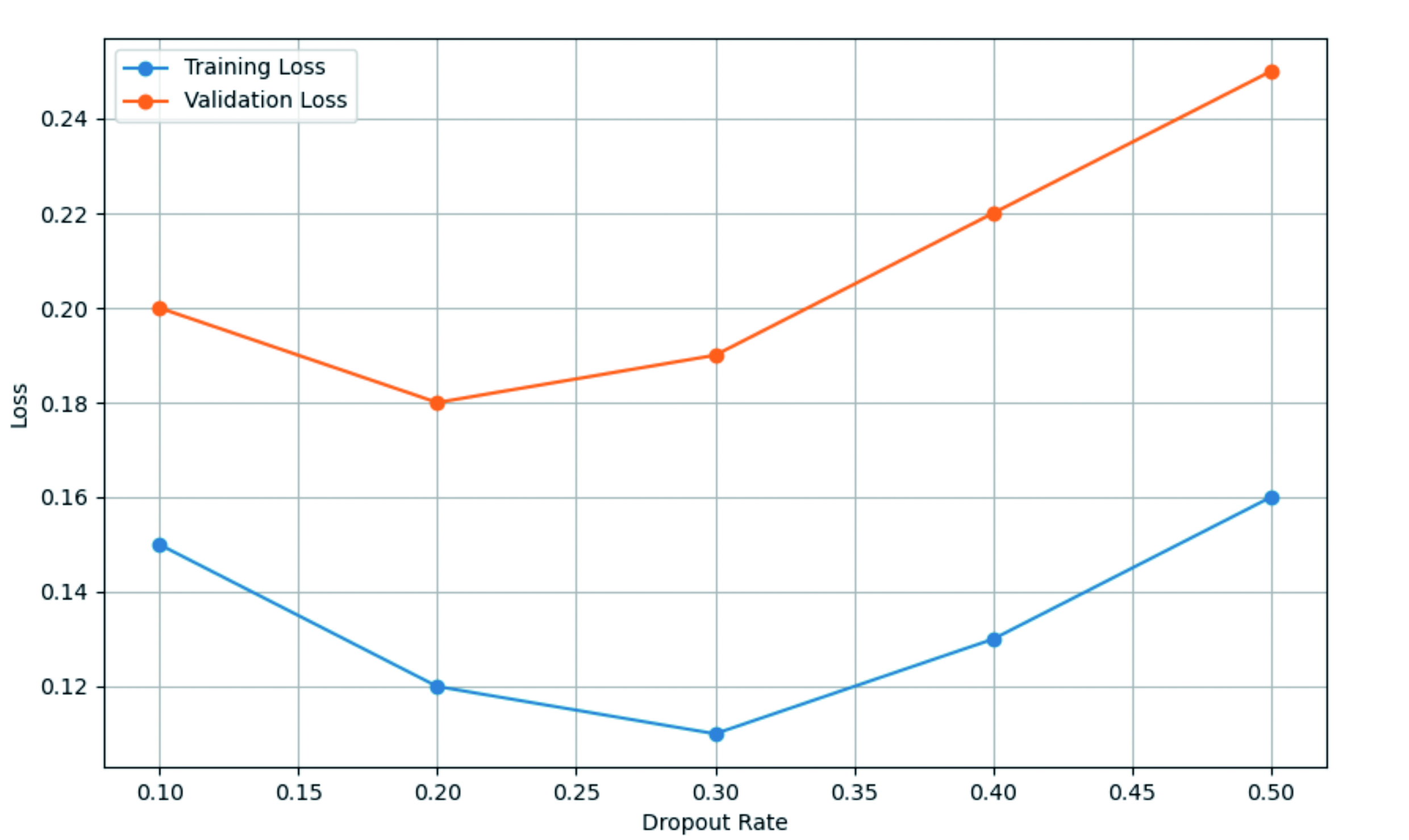

Supplement: S4 Fig — (TIF) [file pone.0342258.s004.tif]

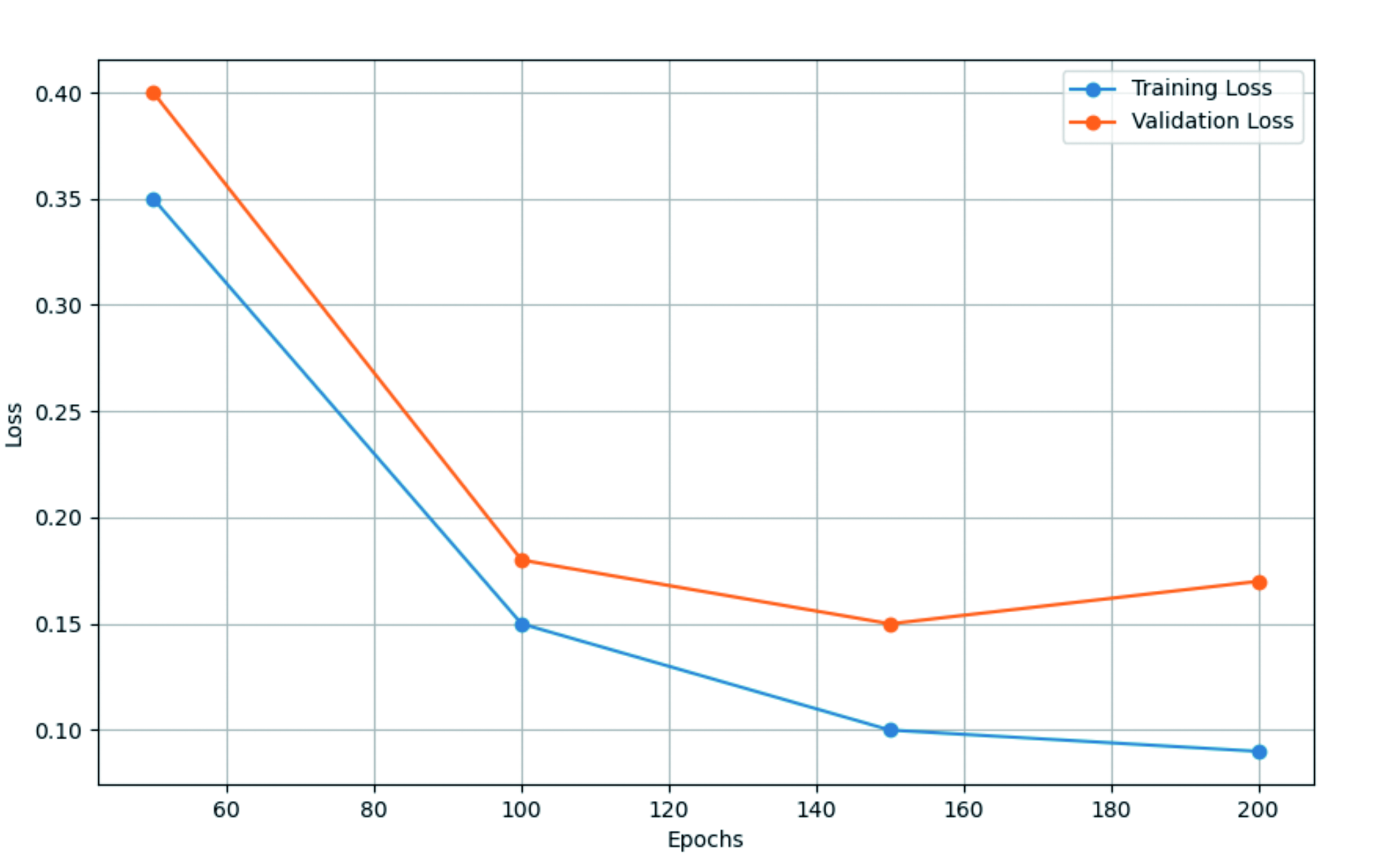

Supplement: S5 Fig — (TIF) [file pone.0342258.s005.tif]

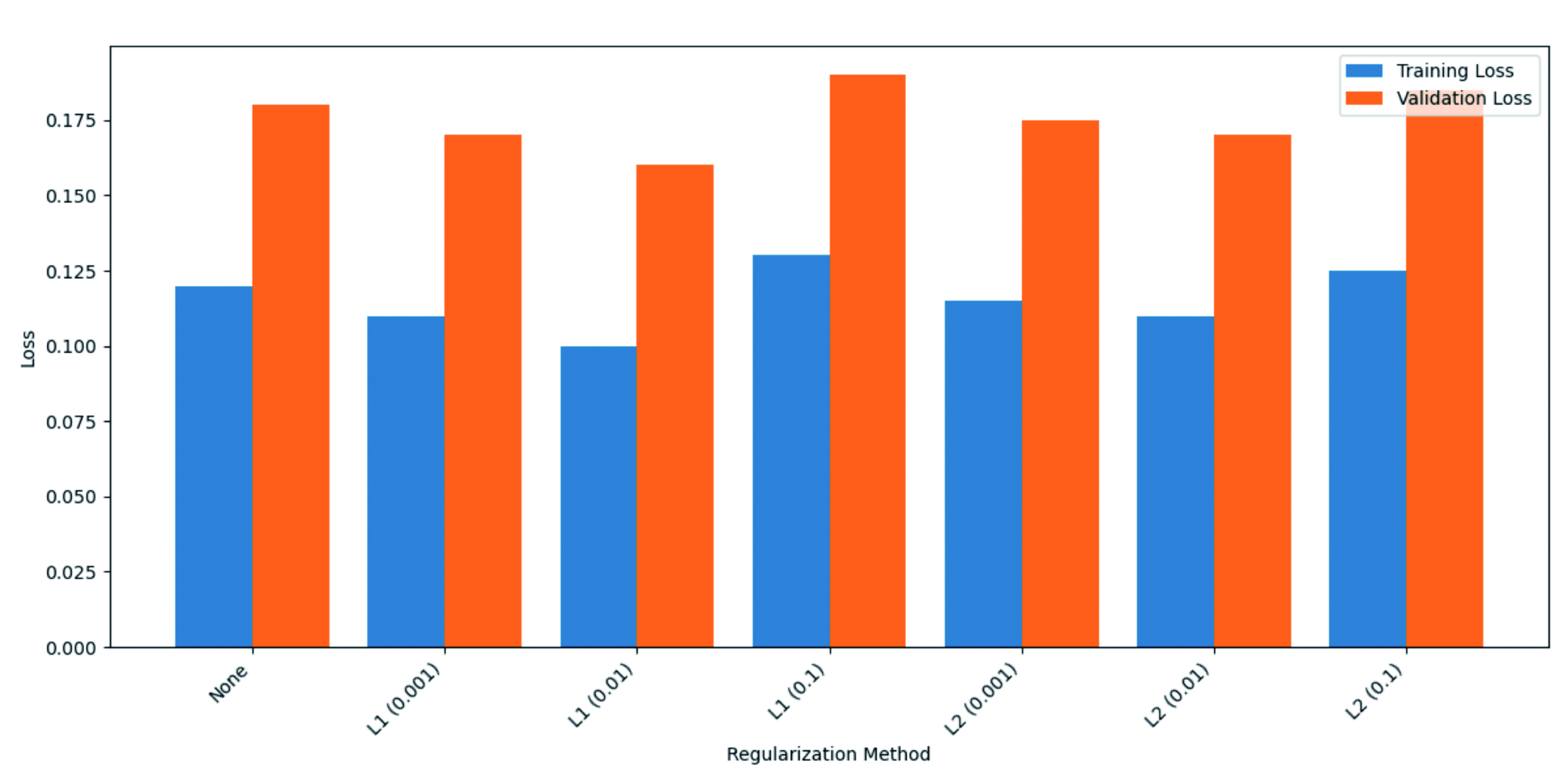

Supplement: S6 Fig — (TIF) [file pone.0342258.s006.tif]

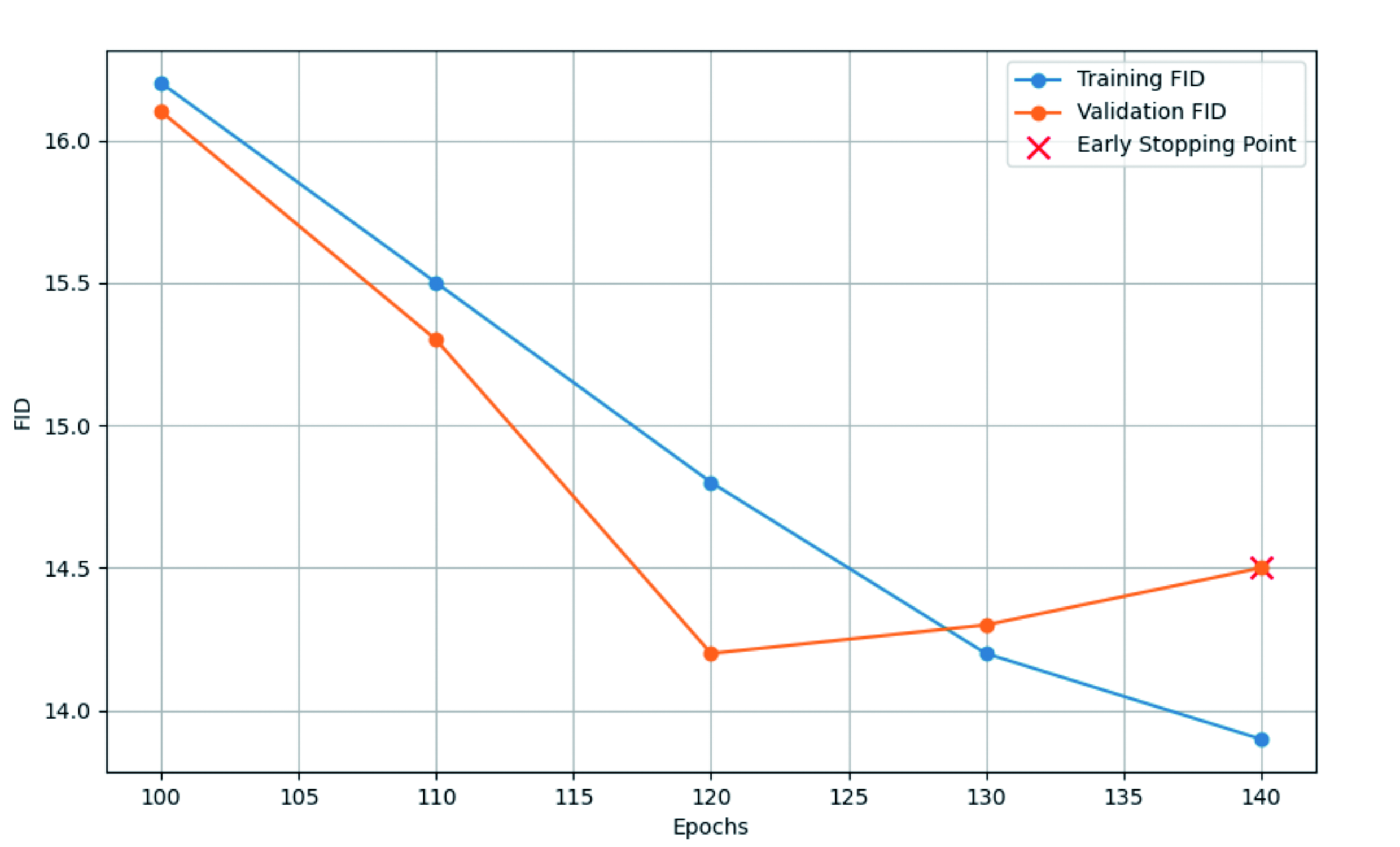

Supplement: S7 Fig — (TIF) [file pone.0342258.s007.tif]

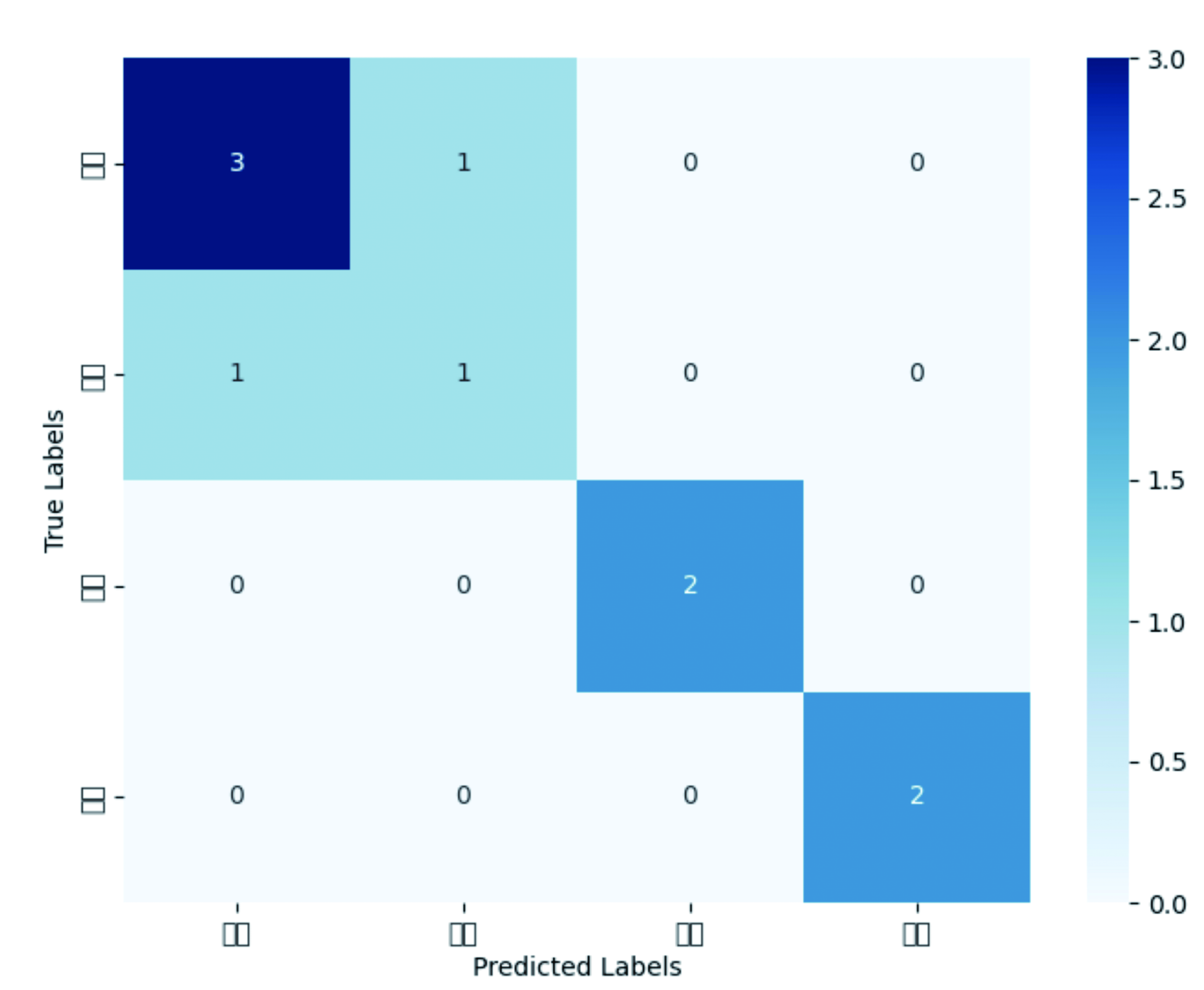

Supplement: S8 Fig — (TIF) [file pone.0342258.s008.tif]

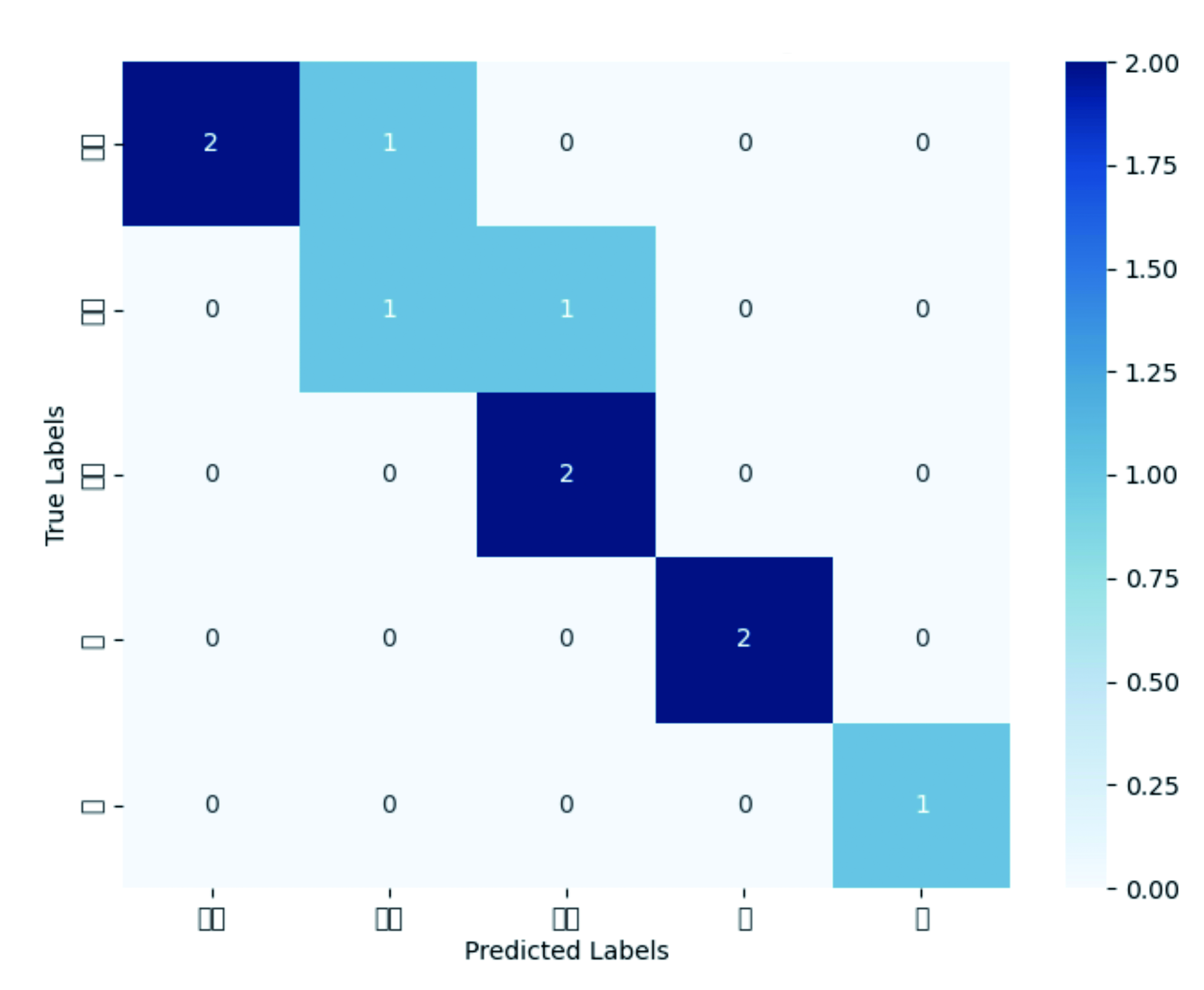

Supplement: S9 Fig — (TIF) [file pone.0342258.s009.tif]
