# Supplementary material for: SDXL model-based optimization for interior design: Data-driven and deep learning methods
Source: PLoS One. 2026 Feb 4;21(2):e0342258. doi: 10.1371/journal.pone.0342258 (PMC12871978; doi:10.1371/journal.pone.0342258)
Supplement: S1 File — (DOCX) [file pone.0342258.s012.docx]

**Appendix A. Core Code of the Crawler Script.**

import scrapy

from scrapy.crawler import CrawlerProcess

import os

from urllib.parse import urljoin

# Set the save directory

SAVE_DIR = "houzz_images"

if not os.path.exists(SAVE_DIR):

os.makedirs(SAVE_DIR)

# Define the spider class

class HouzzSpider(scrapy.Spider):

name = "houzz_spider"

allowed_domains = ["houzz.com"]

start_urls = ["https://www.houzz.com/photos"]

# Parse function

def parse(self, response):

# Find image URLs

images = response.css("img[class*=photo]::attr(src)").getall()

# Download each image

for img_url in images:

if img_url:

# Construct absolute URL

img_abs_url = urljoin(response.url, img_url)

yield scrapy.Request(img_abs_url, callback=self.save_image)

# Handle pagination (if there is pagination)

next_page = response.css("a[class*=next]::attr(href)").get()

if next_page:

next_page_url = response.urljoin(next_page)

yield scrapy.Request(next_page_url, callback=self.parse)

# Callback function to save images

def save_image(self, response):

image_url = response.url

image_name = os.path.basename(image_url.split("?")[0]) # Get file name and remove URL parameters

file_path = os.path.join(SAVE_DIR, image_name)

with open(file_path, "wb") as f:

f.write(response.body)

self.log(f"Image saved: {file_path}")

# Run the spider

if __name__ == "__main__":

process = CrawlerProcess()

process.crawl(HouzzSpider)

process.start()

**Appendix B. Core Code of the Data Cleaning Section.**

import os

import cv2

import imagehash

from PIL import Image

import numpy as np

# Set the image directory path

IMAGE_DIR = "./houzz_images"

CLEAN_IMAGE_DIR = "./cleaned_houzz_images"

if not os.path.exists(CLEAN_IMAGE_DIR):

os.makedirs(CLEAN_IMAGE_DIR)

# Define hash function to detect duplicate images

def is_duplicate(image_path, hash_set):

image = Image.open(image_path)

image_hash = imagehash.average_hash(image)

if image_hash in hash_set:

return True

else:

hash_set.add(image_hash)

return False

# Define image clarity detection function

def is_blurry(image_path):

image = cv2.imread(image_path, cv2.IMREAD_GRAYSCALE)

laplacian_var = cv2.Laplacian(image, cv2.CV_64F).var()

return laplacian_var < 100 # Custom threshold, lower values mean clearer images

# Data cleaning

hashes = set()

for image_file in os.listdir(IMAGE_DIR):

image_path = os.path.join(IMAGE_DIR, image_file)

# Check for duplicate images

if is_duplicate(image_path, hashes):

print(f"Duplicate removed: {image_file}")

continue

# Check if the image is blurry

if is_blurry(image_path):

print(f"Blurry image removed: {image_file}")

continue

# Save cleaned images

cleaned_image_path = os.path.join(CLEAN_IMAGE_DIR, image_file)

os.rename(image_path, cleaned_image_path)

print("Data cleaning completed.")

**Appendix C. Core Code of the Data Labeling Section.**

Import os

import torch

from yolo5 import YOLOv5

from PIL import Image

# Load the pre-trained YOLOv5 model

model = YOLOv5("yolov5x.pt") # Use appropriate weight file

# Set the image directory path

CLEAN_IMAGE_DIR = "./cleaned_houzz_images"

ANNOTATED_DIR = "./annotated_images"

ANNOTATIONS_FILE = os.path.join(ANNOTATED_DIR, "annotations.csv")

if not os.path.exists(ANNOTATED_DIR):

os.makedirs(ANNOTATED_DIR)

# Create an empty annotations file

with open(ANNOTATIONS_FILE, 'w') as f:

f.write("ImageID,DesignStyle,FurnitureType,SpaceFunction\n")

# Automated annotation and manual review

for image_file in os.listdir(CLEAN_IMAGE_DIR):

image_path = os.path.join(CLEAN_IMAGE_DIR, image_file)

image = Image.open(image_path)

# Get YOLO predictions

results = model.predict(image)

# Initial annotation results

design_style = []

furniture_type = []

space_function = []

for result in results:

label = result['label']

if label in ["modern", "vintage", "scandinavian"]: # User-defined categories

design_style.append(label)

if label in ["sofa", "bed", "table", "lamp"]:

furniture_type.append(label)

if label in ["livingroom", "bedroom", "kitchen", "bathroom"]:

space_function.append(label)

# Review and adjust the automatic annotations

print(f"Image: {image_file}")

print(f"Design Styles: {design_style}")

print(f"Furniture Types: {furniture_type}")

print(f"Space Functions: {space_function}")

design_style = input("Confirm or edit Design Styles (comma separated): ").split(',')

furniture_type = input("Confirm or edit Furniture Types (comma separated): ").split(',')

space_function = input("Confirm or edit Space Functions (comma separated): ").split(',')

# Save annotations

with open(ANNOTATIONS_FILE, 'a') as f:

f.write(f"{image_file},{','.join(design_style)},{','.join(furniture_type)},{','.join(space_function)}\n")

# Copy annotated image to the annotated directory

annotated_image_path = os.path.join(ANNOTATED_DIR, image_file)

image.save(annotated_image_path)

**Appendix D. Core Code of the Data Normalization Section.**

import torch

from torchvision import transforms

from PIL import Image

class InteriorDesignAugmenter:

"""

Implements the stochastic data augmentation strategy for Interior Design images.

Includes both geometric and photometric transformations as described in Section 3.2.

"""

def __init__(self, img_size=512):

self.img_size = img_size

# Define the transformation pipeline

self.transform_pipeline = transforms.Compose([

# --- Geometric Transformations ---

# Resize with bicubic interpolation to maintain texture quality

transforms.Resize((self.img_size, self.img_size), interpolation=transforms.InterpolationMode.BICUBIC),

# Random Horizontal Flip (p=0.5):

# Valid for interiors as layout logic is generally symmetric horizontally

transforms.RandomHorizontalFlip(p=0.5),

# Random Rotation (degrees=±10):

# Simulates slight camera tilt without destroying vertical perspective logic

transforms.RandomRotation(degrees=10),

# --- Photometric Transformations ---

# Color Jitter: Randomly change brightness, contrast, and saturation

# Factors are kept low (0.1-0.2) to preserve material authenticity

transforms.ColorJitter(

brightness=0.2,

contrast=0.2,

saturation=0.2,

hue=0.01

),

# --- Normalization ---

# Convert image to tensor and normalize pixel values to [0, 1]

transforms.ToTensor(),

])

def __call__(self, image):

"""

Apply the transformations to an input image.

Args:

image (PIL.Image): The input image (e.g., loaded via PIL).

Returns:

torch.Tensor: The augmented and normalized image tensor [C, H, W].

"""

return self.transform_pipeline(image)

# Example Usage

if __name__ == "__main__":

# Simulate loading a raw image

# raw_image = Image.open("path/to/interior_design_image.jpg")

# For demonstration, create a dummy image

raw_image = Image.new('RGB', (800, 600), color = 'white')

# Initialize the augmenter

augmenter = InteriorDesignAugmenter(img_size=512)

# Process the image

processed_tensor = augmenter(raw_image)

print(f"Output Tensor Shape: {processed_tensor.shape}") # Expected: torch.Size([3, 512, 512])

print(f"Value Range: [{processed_tensor.min():.2f}, {processed_tensor.max():.2f}]")

# Expected: [0.00, 1.00]
